# Temporal Prediction Model-Based Fast Inter CU Partition for Versatile Video Coding

**DOI:** 10.3390/s22207741

**Published:** 2022-10-12

**Authors:** Yue Li, Fei Luo, Yapei Zhu

**Affiliations:** 1 College of Computer Science, University of South China, Hengyang 421001, China; 2 Faculty of Physics and Electronic Information Science, Hengyang Normal University, Hengyang 421002, China

**Keywords:** versatile video coding, QTMT, CU partition, temporal prediction model

## Abstract

Versatile video coding (VVC) adopts an advanced quad-tree plus multi-type tree (QTMT) coding structure to obtain higher compression efficiency, but it comes at the cost of a considerable increase in coding complexity. To effectively reduce the coding complexity of the QTMT-based coding unit (CU) partition, we propose a fast inter CU partition method based on a temporal prediction model, which includes early termination QTMT partition and early skipping multi-type tree (MT) partition. Firstly, according to the position of the current CU, we extract the optimal CU partition information of the position corresponding to the previously coded frames. We then establish a temporal prediction model based on temporal CU partition information to predict the current CU partition. Finally, to reduce the cumulative of errors of the temporal prediction model, we further extract the motion vector difference (MVD) of the CU to determine whether the QTMT partition can be terminated early. The experimental results show that the proposed method can reduce the inter coding complexity of VVC by 23.19% on average, while the Bjontegaard delta bit rate (BDBR) is only increased by 0.97% on average under the Random Access (RA) configuration.

## 1. Introduction

With the rapid development of display technology, new video formats such as Ultra-High Definition (UHD), High Dynamic Range (HDR), and 360 videos have provided improved visual experiences. However, their considerable data volume is a challenge for video storage and real-time transmission. To compress different emerging video formats more efficiently, the Joint Video Experts Team (JVET) formulated a new generation of video compression standards in July 2020, namely, Versatile Video Coding (VVC) [1].

Compared with High-Efficiency Video Coding (HEVC) [2], VVC reduces the coding rate by more than 30% while maintaining the same subjective video quality [3]. This is because many advanced coding techniques are introduced in VVC. To further remove the spatial coding redundancy, VVC proposes new technologies, such as extending the 35 intra prediction modes in HEVC to 67 [4], multiple reference line (MRL) prediction [5], and intra sub-partitioning (ISP) [6]. Advanced techniques such as affine motion compensation prediction, adaptive motion vector resolution, and bidirectional optical flow have been proposed to further remove the temporal coding redundancy of VVC [7]. In addition, to adapt to the different textures and motion characteristics of coding regions, VVC adopts a new coding structure, the quad-tree plus multi-type tree (QTMT) partition structure [8], which can flexibly divide the coding unit (CU) into square and rectangular regions of different sizes. Although these new coding techniques can effectively improve the compression efficiency of intra and inter frames, they also drastically increase the coding complexity of VVC intra and inter frames, which has become a major obstacle to the deployment of VVC real-time applications. In particular, the flexible QTMT partition structure process accounts for considerable coding complexity [9]. Therefore, it is necessary to ensure that fast decisions can be made in the QTMT partition process to greatly reduce the coding complexity of VVC.

The QTMT partition structure is an extension of the quad-tree (QT) partition structure in HEVC. In HEVC, the QT partition also occupies a considerable amount of coding complexity. To effectively reduce the coding complexity of HEVC, researchers have proposed many fast QT partition methods [10,11,12,13,14,15,16]. These methods can be mainly classified into two categories: correlation-based methods and learning-based methods. For example, in [12], a fast QT partition method is proposed by using the spatial and temporal correlation between the current coding CU and the coded CUs. In [15], to accelerate the QT partition decision, the prediction model is trained by the deep learning method to predict the optimal QT partition in advance. However, these fast QT partition methods for HEVC cannot be directly used in the QTMT partition of VVC, because the multi-type tree (MT) partition is added to the QTMT partition structure, which makes the CU partition more flexible. In addition, many new coding techniques are applied to the QTMT partition structure, which results in the optimal CU partition in VVC and HEVC being different in the same coding regions.

To reduce the coding complexity of the intra VVC encoder, many fast CU decision methods [17,18,19,20,21,22,23,24,25,26,27,28,29] have proposed the acceleration of intra QTMT partition. For example, in [19], the authors propose a directional gradient-based early termination CU partition method in which four directional gradients (horizontal, vertical, 45, and 135) of CUs are extracted separately to represent the relationship between the optimal CU partition and the texture characteristics of the CU. In [23], a fast decision method for QTMT partition with a cascaded decision structure composed of decision trees is proposed. In addition, the authors in [25,26,27,28,29] adopted a deep-learning-based approach for fast QTMT partition decisions. However, these fast CU decision methods for intra QTMT partition cannot be directly used for inter QTMT partition, because the optimal intra CU partition of a CTU is mainly related to the texture characteristics of the CU under the intra coding. However, the optimal inter CU partition of a CTU is not only related to the CU’s texture characteristics but also related to the motion properties of the CU. Therefore, it is necessary to propose a fast inter QTMT partition decision method to reduce the inter coding complexity of VVC.

Researchers have proposed many fast CU partition decision methods for reducing the inter coding complexity of VVC [30,31,32,33]. In [30], the difference between three frames is extracted to indicate that the CU is a motion block or a static block. If the CU is a static block, the CU is terminated for further partition early to reduce the complexity of inter coding. In [31], three binary classifiers are established by using the random forest to accelerate the inter CU partition decision. In addition, the tunable risk interval is defined to obtain different coding complexity reductions. In [32], combining the luminance, residual, and bidirectional motion information of the CU as the input information of a convolutional neural network (CNN), a multi-information fusion convolutional neural network (MF-CNN)-based early inter CU partition termination method is proposed. In addition, the Merge mode of the CU is terminated early using the residual information of the CU to further reduce the coding complexity of the inter CU. In [33], a multi-level tree convolutional neural network (MLT-CNN) is proposed for a fast CU partition decision. First, the order of the decision process of CU is split into four levels: no partition–partition, QT partition–MT partition, binary tree (BT) partition–ternary tree (TT) partition, and vertical partition–horizontal partition. The MLT-CNN is then used to predict how the CU is split in each level. However, the temporal correlation of CU partition is not fully explored in VVC inter coding. Although many methods have proposed utilizing temporal correlation for fast QT-based CU partition decisions in HEVC, they cannot be directly used in the new QTMT-based CU partition. Therefore, it is possible to explore fast QTMT partition decisions based on temporal correlations. In this paper, we propose a temporal prediction model-based approach to predict CU partition to reduce the inter coding complexity of VVC. The main contributions are summarized as follows.

- According to the partition of the encoded CU at the corresponding position of the current CU, we established a temporal prediction model to predict the optimal CU partition.
- To reduce prediction errors, we further extract the motion vector information of the CU to determine whether the CU partition can be terminated early.
- Compared with deep learning methods, our proposed method does not require the establishment of additional large datasets for training and does not require additional complex training to obtain decision parameters.

We organize the remainder of this paper as follows. Section 2 presents the motivation and statistical analyses. Section 3 presents the proposed fast inter CU partition method of VVC based on the temporal prediction model. Section 4 presents the experimental results. The conclusion is in Section 5.

## 2. Motivation and Statistical Analyses

**Figure 1:**
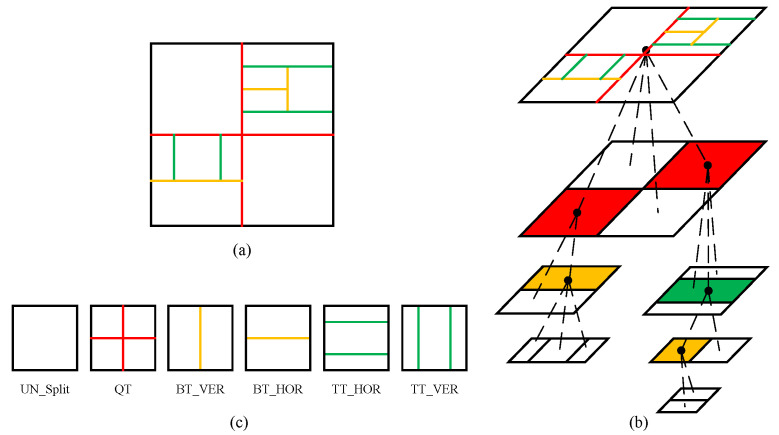
An example of optimal CU partition of a CTU. (**a**,**b**) denote the optimal CU partition, (**c**) presents six types of CU partition.

A new QTMT-based CU partition structure is introduced in VVC to further improve the compression efficiency, and its flexible and changeable partition method is more suitable for video content with different textures and motion characteristics. In the VVC coding standard, a coding image is firstly split into multiple Coding Tree Units (CTUs), the default size of which is 128 × 128. Afterwards, the CTU is used as the root node for further hierarchical partition. The blocks obtained by the partition process are called Coding Units (CUs). A CTU can contain one CU or be recursively split into smaller square CUs or rectangular CUs, where a square CU can be obtained by QT partition, while a rectangular CU is obtained by MT partition. MT includes horizontal BT, vertical BT, horizontal TT, and vertical TT. Figure 1 shows an example of optimal CU partition of a CTU, and the six partition methods supported by the CU. In order to obtain the optimal CTU partition, a 128 × 128 CTU will first perform a top-to-bottom multi-level hierarchical partition process until the minimum CU size is 4 × 4, and each CU needs to calculate the rate-distortion cost (RD cost). CU pruning is then performed in a bottom-up manner, and the CUs with the smallest RD cost are combined to obtain the optimal CTU partition. This method of obtaining the optimal CU partition through the brute-force search process will lead to a considerable increase in the coding complexity of VVC. Although a CTU needs to traverse CUs of different sizes, only a few CUs of different sizes are selected in the optimal CTU partition. If the optimal CU partition in the CTU can be predicted early, and the RD cost calculation of the remaining CUs can be skipped, the coding complexity of VVC can be effectively reduced.

### 2.1. The Distribution of Different CU Sizes

In VVC inter coding, a larger CU size is usually selected as the optimal CU partition for background or slow-moving regions, while a smaller CU size is usually selected as the optimal CU for regions with complex motion regions. To represent the distribution of optimal CU sizes in VVC inter coding, four video sequences with different motion characteristics are tested, including “MarketPlace” (1920 × 1080), “FourPeople” (1280 × 720), “BasketballDrill” (832 × 480), and “BasketballPass” (416 × 240). Test conditions are shown in Table 1. To simplify the statistical distribution of different CU sizes, we divide all optimal CU sizes into five non-overlapping categories, L64, L32, L16, L8, and L4, which correspond to the width and height of the CUs that are all greater than or equal to 64, 32, 16, 8, and 4, respectively. For example, an optimal CU with a width of 64 and a height of 32 will be classified as L32. The optimal CU size distributions for video sequences under different QPs are given in Table 2. From Table 2 we can observe the following points:

(1) For all video sequences, the average proportions of L64, L32, L16, L8, and L4 are 58.33%, 18.26%, 13.35%, 7.45%, and 2.61%, which indicates that the larger-size CUs are selected as optimal CUs for most regions. Under the same QP, as the CU size decreases, its proportion becomes smaller.

(2) With the increase of QP, the proportion of CUs in the L64 becomes larger, and the proportion of CUs in the L8 and L4 becomes smaller. When QP = 37, the maximum proportion of CU under the L64 is 89.62%, and the minimum proportion is 53.72%. The largest proportions of CUs in the L8 and L4 are only 4.89% and 1.81%. When QP = 22, the maximum proportions of CUs under L8 and L4 are 16.84% and 8.26%. This indicates that different QPs have a significant impact on the optimal CU selection for the same coding regions.

(3) For the sequence “FourPeople” containing large background regions, the proportion of CUs in the L64 exceeds 70.72%, and the proportion of CUs in the L4 is less than 2.15%. For the sequence “BasketballDrill” with complex motion regions, and the sequences “MarketPlace” and “BasketballPass” containing dynamic backgrounds and camera movements, the sum of L64 and L32 is larger. This shows that, for sequences with different motion characteristics, the distribution of the optimal CU size is different, and a larger CU size is usually selected as the optimal CU for background or slow-moving regions.

From the above observations, it can be concluded that, for different video sequences, if the CU partition can be accurately predicted in advance, it will greatly reduce the coding complexity of VVC.

### 2.2. Temporal Correlation of CUs

**Figure 2:**
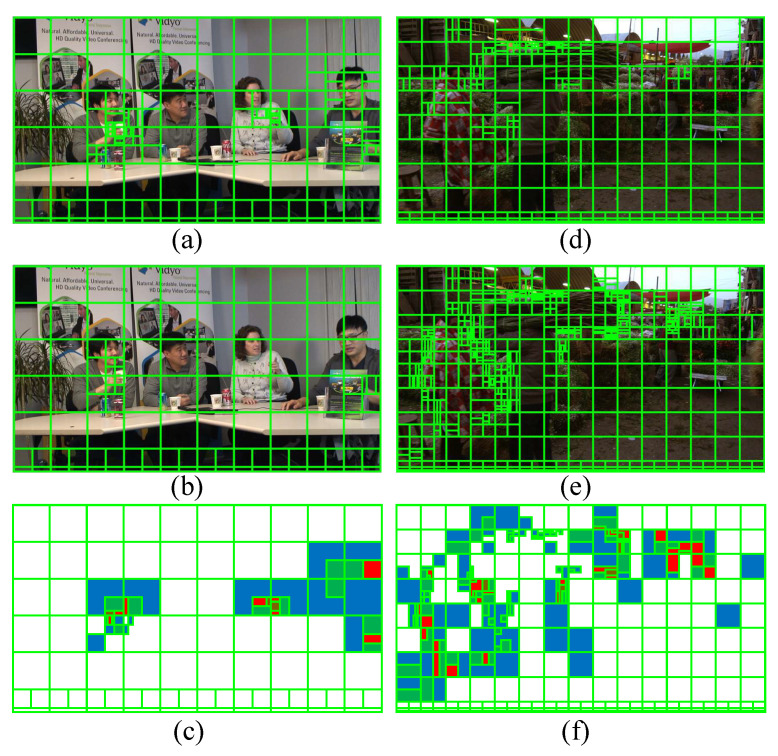
Optimal CU partition of two successive frames of sequences “FourPeople” and “MarketPlace” and the absolute differences of CU depth among frames. (**a**,**b**,**d**,**e**) denote the original optimal CU partition. (**c**,**f**) represent the absolute difference between (**a**,**b**), and the absolute difference between (**d**,**e**), respectively.

Since the content between adjacent frames is very similar, there is a high probability that the corresponding regions between adjacent frames have the same optimal CU partition. To explore the correlation of optimal CU partition between adjacent frames in the same regions, Figure 2 presents the optimal CU partition for sequences “FourPeople” and “MarketPlace” under RA configuration. In VVC, the largest CU size of 128 × 128 corresponds to a CU depth of 0. As the CU is split into smaller sub-CUs, its corresponding depth is increased by 1. Figure 2c,f show the absolute difference of CU depth between the 5th and 6th frames. In Figure 2c,f, the four colors white, blue, green, and red represent the absolute difference of zero, one, two, and three, respectively, and black represents the depth difference greater than or equal to four. We can further observe that most background regions are marked with white or blue, which indicates that the CU depth correlation between adjacent frames is very high.

To analyze the CU depth difference ($d_{\psi }$) between adjacent frames, $d_{\psi }$ is defined as follows:

(1) $$d_{\psi }=\left|{\mathrm{CU}}_{\psi }^{i}-{\mathrm{CU}}_{\psi }^{i+1}\right|,\psi \in \left\{\mathrm{QTMT},\mathrm{QT},\mathrm{MT}\right\}$$

where ${\mathrm{CU}}_{\psi }^{i}$ and ${\mathrm{CU}}_{\psi }^{i+1}$ represent the CU depth of the i-th frame and the *i* + 1-th frame at the same position. The test sequences include “FourPeople” and “MarketPlace”, and the test conditions are shown in Table 1. Table 3, Table 4 and Table 5 represent the QTMT depth difference, the QT depth difference, and the MT depth difference of adjacent frames, respectively. It can be seen in Table 3, Table 4 and Table 5 that the average percentages of depth differences of QTMT, QT, and MT that are less than or equal to 1 are 77.5%, 95.1%, and 85.8%, which indicates that the CU depth difference between adjacent frames is very small. Therefore, the CU depth of adjacent coded frames can be used to predict the optimal CU depth of the current coding frame.

## 3. The Proposed Fast Inter CU Partition Method

In VVC, the optimal CU partition of a CTU is selected by traversing all partition sizes. Therefore, if the most probable depth of the current coding CU can be accurately determined in advance, a large amount of coding complexity can be reduced without affecting the coding quality. This section is mainly divided into two parts. Firstly, a temporal prediction model that predicts the most probable depth of the CU is established. Secondly, a fast CU partition decision method is proposed based on the temporal prediction model.

### 3.1. The Temporal Prediction Model

**Figure 3:**
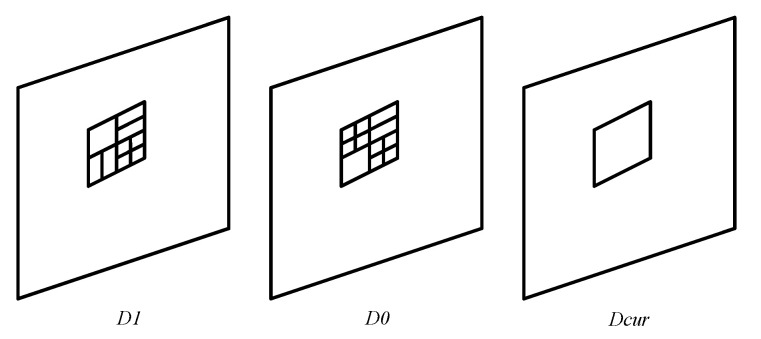
An example of current coding frame and adjacent coded frames.

According to the analysis in Section 2.2, it can be concluded that adjacent frames have very similar texture content, which leads to a strong correlation between the optimal CU depth (CU partition) at the same positions. Therefore, based on this heuristic, we can utilize the optimal CU depth of previously coded frames to predict the optimal CU depth of the current coding frame. Figure 3 shows an example of a CU in the current coding frame and a corresponding position CU in the coded frames, $D_{0}$ and $D_{1}$ respectively represent the previously coded frame, and Dcur represents the current coding frame. In this paper, a temporal prediction model is established to predict the current CU depth according to the temporal correlation of the CU, which is defined as follows:

(2) $$D_{p}=\left(D_{0}+D_{1}+1\right)/2$$

(3) $$D_{0}=\arg\max\left\{D_{0}^{w\times h}\right\}$$

(4) $$D_{1}=\arg\max\left\{D_{1}^{w\times h}\right\}$$

where $D_{0}$ and $D_{1}$ respectively represent the maximum depth of the CU in the previously encoded frames, and *w* and *h* denote the width and height of the CU. Since the optimal depth of the CU has a strong correlation with the QP, the coded frames are the nearest adjacent frames with the same or less QP as the current coding frame.

### 3.2. Terminating QTMT Partition Early Based on the Temporal Prediction Model

(a) Terminating the QT partition early:

As shown in Figure 1, the optimal depth of a CU in VVC coding may include both QT depth and MT depth. Therefore, early QT depth and MT depth are both decisions in this paper. According to the temporal prediction model in Section 3.1, the optimal QT depth model for the prediction of the current CU is expressed as follows:

(5) $$QT_{p}=\left(QT_{0}+QT_{1}+1\right)/2$$

(6) $$QT_{0}=\arg\max\left\{{\mathrm{QT}}_{\mathrm{CU}}^{0}\right\}$$

(7) $$QT_{1}=\arg\max\left\{{\mathrm{QT}}_{\mathrm{CU}}^{1}\right\}$$

where ${\mathrm{QT}}_{0}$ and ${\mathrm{QT}}_{1}$ respectively represent the maximum QT depth of the coded CU in the previous frames. According to Equations (5)–(7), early QT depth decision is defined as follows:

(8) $${\mathrm{CU}}_{\mathrm{QT}}\in \left\{\begin{array}{c} \mathrm{un}\_\mathrm{split}\;\;\;\;\;\;\;\;\;\;\;\;\;\;\;\;\;\;\;\;\;\;\;QT_{\mathrm{cur}}\geq QT_{p}+{\mathrm{Th}}_{\mathrm{QT}}\\ \mathrm{split}\;\;\;\;\;\;\;\;\;\;\;\;\;\;\;\;\;\;\;\;\;\;\;\;\;\;\;\;\;\;\;\;\;\;\;\;\;\;\;\;\;\;\mathrm{otherwise} \end{array}\right.$$

where ${\mathrm{QT}}_{\mathrm{cur}}$ represents the QT depth of the current CU, ${\mathrm{QT}}_{p}$ denotes the prediction QT depth of the current CU according to the temporal prediction model, and ${\mathrm{Th}}_{\mathrm{QT}}$ is an adaptive threshold. If ${\mathrm{QT}}_{\mathrm{cur}}$ is less than ${\mathrm{QT}}_{p}$ + ${\mathrm{Th}}_{\mathrm{QT}}$, the current CU needs to perform the QT partition. Otherwise, the QT partition is terminated early.

(b) Terminating the MT partition early:

Similar to the optimal QT depth model for prediction of the current CU, according to the temporal prediction model established in Section 3.1, the optimal MT depth model for the prediction of the current CU is expressed as follows:

(9) $$MT_{p}=\left(MT_{0}+MT_{1}+1\right)/2$$

(10) $$MT_{0}=\arg\max\left\{{\mathrm{MT}}_{\mathrm{CU}}^{0}\right\}$$

(11) $$MT_{1}=\arg\max\left\{{\mathrm{MT}}_{\mathrm{CU}}^{1}\right\}$$

where ${\mathrm{MT}}_{0}$ and ${\mathrm{MT}}_{1}$ respectively represent the maximum depth of the BT depth and the TT depth of the coded CU in the previous frames. According to Equation (9)–(11), the early MT depth decision is defined as follows:

(12) $${\mathrm{CU}}_{\mathrm{MT}}\in \left\{\begin{array}{c} \mathrm{un}\_\mathrm{split}\;\;\;\;\;\;\;\;\;\;\;\;\;\;\;\;\;\;\;\;\;\;\;MT_{\mathrm{cur}}\geq MT_{p}+{\mathrm{Th}}_{\mathrm{MT}}\\ \mathrm{split}\;\;\;\;\;\;\;\;\;\;\;\;\;\;\;\;\;\;\;\;\;\;\;\;\;\;\;\;\;\;\;\;\;\;\;\;\;\;\;\;\;\;\mathrm{otherwise} \end{array}\right.$$

where ${\mathrm{MT}}_{\mathrm{cur}}$ represents the MT depth of the current CU, ${\mathrm{MT}}_{p}$ denotes the prediction MT depth of the current CU according to the temporal prediction model, and ${\mathrm{Th}}_{\mathrm{MT}}$ is an adaptive threshold. If ${\mathrm{MT}}_{\mathrm{cur}}$ is less than ${\mathrm{MT}}_{p}$+${\mathrm{Th}}_{\mathrm{MT}}$, the current CU needs to perform the MT partition. Otherwise, the MT partition is terminated early.

### 3.3. Prediction Error Reduction Based on Encoding Information

In Section 3.1 and Section 3.2, although the prediction depth can be used for early termination of the current CU depth, the process of early termination may lead to the accumulation of errors in depth prediction. This is because, on the one hand, the optimal depth of the current CU is predicted based on the previously coded frames, and when the next frame is coding, the CU depth of the current coding frame is used to predict the optimal CU depth of the next frame. On the other hand, when scene switching occurs between adjacent frames, directly using the CU depth information of the previous frame will not be able to accurately determine the optimal CU depth of the current frame. In order to effectively solve the above problems, the coding information of the CU is introduced, namely, motion vector difference (MVD). In inter coding, the MVD of the CU can well represent the relative motion of the CU. When the MVD of the CU is 0, the CU is in the static region or the region with the same motion direction as the adjacent CUs, and the CUs in these regions usually do not need to be further split. In order to explore the relationship between the MVD of CU and CU partition, we counted the probability that the MVD of CU is 0 when the encoding frame is the optimal CU partition. The experimental setting conditions are the same as in Section 2.1. Table 6 shows the probability that the MVD of a CU is 0. It can be seen in Table 6 that the MVD of more than 91.3% of the CUs on average is 0. Therefore, in this paper, we combine the MVD of the CU for early QTMT depth decision. Equations (8) and (12) for early QTMT depth decisions are modified as follows:

(13) $${\mathrm{CU}}_{\mathrm{QT}}\in \left\{\begin{array}{c} \mathrm{un}\_\mathrm{split}\;\;\;\;\;\;\;\;\;\;\;\;\;\;\;\;\;\;\;\left(QT_{\mathrm{cur}}\geq QT_{p}+{\mathrm{Th}}_{\mathrm{QT}}\right)\&\left(\mathrm{MVD}\neq 0\right)\\ \mathrm{split}\;\;\;\;\;\;\;\;\;\;\;\;\;\;\;\;\;\;\;\;\;\;\;\;\;\;\;\;\;\;\;\;\;\;\;\;\;\;\;\mathrm{otherwise} \end{array}\right.$$

(14) $${\mathrm{CU}}_{\mathrm{MT}}\in \left\{\begin{array}{c} \mathrm{un}\_\mathrm{split}\;\;\;\;\;\;\;\;\;\;\;\;\;\;\;\;\;\;\;\;\;\left(MT_{\mathrm{cur}}\geq MT_{p}+{\mathrm{Th}}_{\mathrm{MT}}\right)\&\left(\mathrm{MVD}\neq 0\right)\\ \mathrm{split}\;\;\;\;\;\;\;\;\;\;\;\;\;\;\;\;\;\;\;\;\;\;\;\;\;\;\;\;\;\;\;\;\;\;\;\;\;\;\;\;\;\;\mathrm{otherwise} \end{array}\right.$$

(15) $$\mathrm{MVD}=\mathrm{MV}D_{\mathrm{hor}}+\mathrm{MV}D_{\mathrm{ver}}$$

where “&” is the logical and. ${\mathrm{MVD}}_{\mathrm{hor}}$ and ${\mathrm{MVD}}_{\mathrm{ver}}$ represent the motion vector difference in the horizontal and vertical directions of the CU, respectively.

### 3.4. Skipping TT Partition Early Based on the Temporal Prediction Model

Since the MT depth may only include BT partition or less TT partition, to further reduce the coding complexity of the MT partition, only BT partition can be performed at a certain MT depth, and the TT partition can be skipped early. According to Equation (9), the early skipping of the TT partition decision is defined as follows:

(16) $$CU_{\mathrm{TT}}\in \left\{\begin{array}{c} \mathrm{skip}\;\;\;\;\;\;\;\;\;\;\;\;\;\;\;\;\;\;\;\;\;\;\;\;\;\;\;\;\;\;\;\;\;MT_{\mathrm{cur}}<MT_{p}-{\mathrm{Th}}_{\mathrm{MT}}\\ \mathrm{un}\_\mathrm{skip}\;\;\;\;\;\;\;\;\;\;\;\;\;\;\;\mathrm{otherwise} \end{array}\right.$$

If ${\mathrm{MT}}_{\mathrm{cur}}$ is less than ${\mathrm{MT}}_{p}$-${\mathrm{Th}}_{\mathrm{MT}}$, the current CU needs to skip the TT partition. Otherwise, the TT partition is performed.

### 3.5. Proposed Overall Method

**Figure 4:**
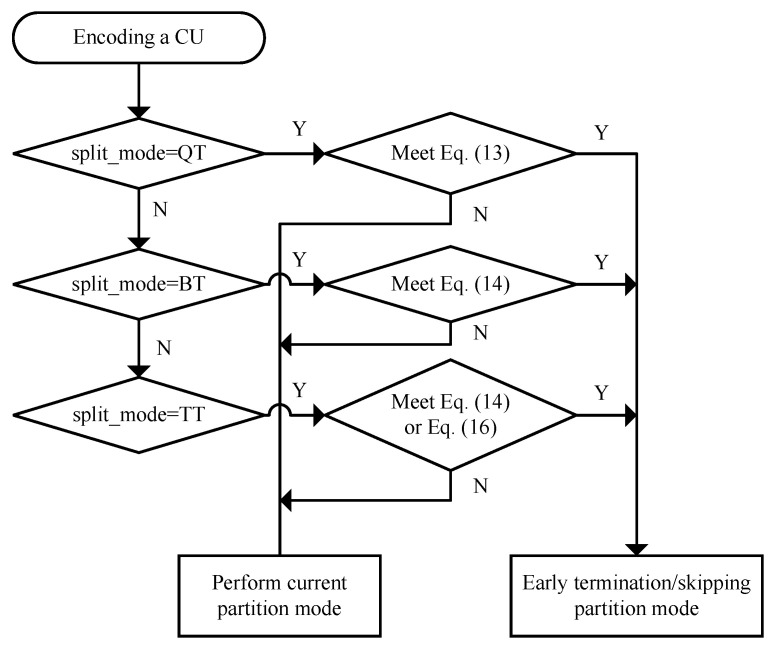
The flowchart of the proposed method.

The basic idea of the proposed fast inter CU partition method of VVC is to use the optimal CU partition information at the corresponding position of the encoded frames to predict the current CU partition. Figure 4 shows the flowchart of the proposed method, and the specific process is summarized as follows:

(1) Start inter CU coding and calculate the RD cost of the CU.
(2) Calculate $QT_{p}$ and $MT_{p}$ according to the temporal prediction model.
(3) When the partition mode is QT, if Equation (13) is satisfied, terminate the QT partition early; otherwise, perform QT partition.
(4) When the partition mode is BT, if Equation (14) is satisfied, terminate the BT partition early; otherwise, perform BT partition.
(5) When the partition mode is TT, if Equation (14) or Equation (16) is satisfied, terminate/skip the TT partition early; otherwise, perform TT partition.

## 4. Experimental Results and Analyses

### 4.1. Test Conditions

To effectively evaluate the encoding performance of the proposed method, the Bjontegaard Delta Bit Rate (BDBR) [34] and coding complexity saving are adopted, where coding time saving is defined as follows:

(17) $$TS=\frac{T_{ori}-T_{pro}}{T_{ori}}$$

where $T_{ori}$ represents the coding time of the original reference software, and $T_{pro}$ denotes the coding time after integrating the proposed method into the reference software. The experimental reference software version is VTM-6.0, the sequences used for the experimental test are the common test sequences [35], the encoding configuration is set to Random Access (RA), and four quantization parameters (QPs) (22, 27, 32, and 37) are used in the encoding. ${\mathrm{Th}}_{\mathrm{QT}}$ and ${\mathrm{Th}}_{\mathrm{MT}}$ are set to 2 and 1, respectively.

### 4.2. Experimental Results

Table 7 presents the coding performance of the early TT partition skipping method, the early QTMT partition termination method, and the proposed overall method. It can be observed in Table 7 that skipping the TT partition early can reduce the coding time by 15.48% on average and increase the BDBR by 0.61%. Early QTMT partition termination can reduce the coding time by 6.14% on average and increase the BDBR by 0.4%. Compared with the early QTMT partition termination method, the early TT partition skipping method can reduce more coding time. The reason for this is that the early termination conditions of the early QTMT partition termination method are stricter, resulting in fewer QTMT partitions being terminated early. The proposed overall method can reduce 8.88–42.08% of the encoding time, with an average saving of 23.19%, and BDBR increases by 0.29–1.98%, while the average increase is only 0.97%. The largest coding time saving is the sequence “Campfire”, with a 42.08% reduction in coding time and a 1.98% increase in BDBR. The smallest coding time saving is the sequence “Johnny”, with an 8.88% reduction in encoding time and a 0.29% increase in BDBR.

**Figure 5:**
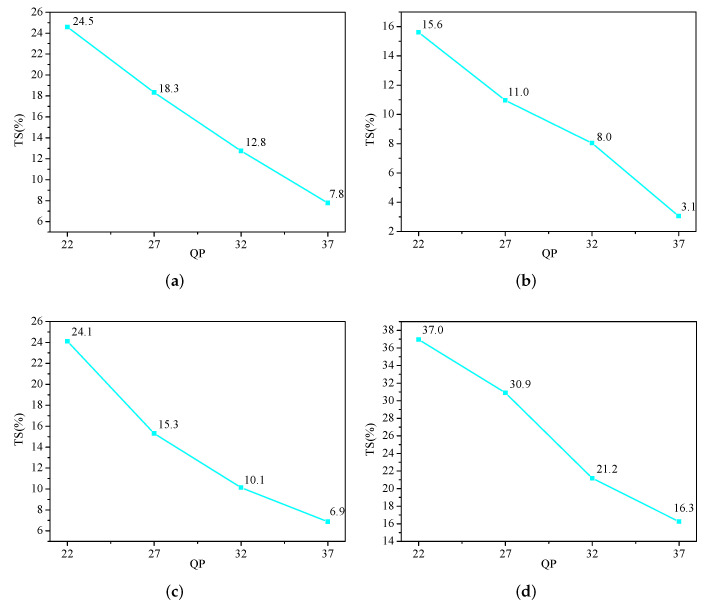
Encoding time saving under different QPs. (**a**) BasketballPass, (**b**) FourPeople, (**c**) MarketPlace, and (**d**) BasketballDrill.

Figure 5 presents the time savings of four sequences under different QPs. It can be seen in Figure 5 that the time saving is smaller as the QP increases, which indicates that the proposed method can save more time under a smaller QP. This is because, as shown in Table 2, when the coding QP is small, a larger depth of the sequence (a smaller CU size) is selected as the optimal depth, and the proposed method can skip the calculation of the smaller depth early. Therefore, the proposed method can save more coding time when the QP is small.

Table 8 presents a coding performance comparison between the proposed method and the current state-of-the-art methods [30,32]. For a fair comparison, the method in [32] only includes the fast CU partition decision method and does not include the early merge mode decision method. Table 8 shows that the method in [30] reduces coding time by 8.29–38.53%, with an average saving of 26.56%, and the BDBR is increased by 0.31–10.10%, with an average increase of 3.29%. Using the method in [32], the coding time is reduced by 9.69–42.90%, with an average saving of 24.83%, and the BDBR is increased by 0.84–5.59%, with an average increase of 2.52%. Compared to these methods [30,32], the proposed method can further reduce BDBR by 2.32% and 1.55%, respectively, on average, with similar coding time savings. In addition, the proposed method has a maximum BDBR increase of 1.98%, which is much less than the 10.10% of the method in [30] and the 5.59% of the method in [32].

## 5. Conclusions

In this paper, a fast inter CU partition decision method is proposed for VVC based on a temporal prediction model to reduce inter coding complexity. First, a temporal prediction model is established using the temporal correlation of CUs. Afterwards, the early termination and early skipping of CU partition are performed according to the temporal prediction model. Finally, to reduce prediction errors, the MVDs of the CUs are jointly used for fast CU partition decisions. Experimental results show that the coding time saved by the proposed fast CU partition method ranges from 8.88% to 42.08%, and the BDBR increases from 0.29% to 1.98%. The comparison of experimental results shows that the BDBR can be further reduced with similar encoding time savings compared to the state-of-the-art methods [30,32].

## Figures and Tables

**Table 1:** Test conditions.

Test platform	VTM6.0
Configuration	Random Access (RA)
CTU size	128 × 128
Quantization Parameter (QP)	22, 27, 32, 37
Number of coded frames	32

**Table 2:** Optimal CU size distribution under different QPs (unit: %).

Sequences	QP	L64	L32	L16	L8	L4
	22	36.67	25.26	23.59	12.24	2.24
MarketPlace	27	59.74	17.89	13.82	7.39	1.16
(1920 × 1080)	32	69.84	14.85	10.24	4.50	0.57
	37	78.29	11.52	7.31	2.63	0.25
	22	70.72	10.82	10.48	5.83	2.15
FourPeople	27	82.71	6.36	6.62	3.07	1.24
(1280 × 720)	32	87.00	4.49	5.49	2.15	0.87
	37	89.62	3.50	4.81	1.52	0.55
	22	33.65	23.87	17.38	16.84	8.26
BasketballDrill	27	42.37	22.96	17.54	12.75	4.38
(832 × 480)	32	50.26	22.45	16.52	8.81	1.96
	37	58.21	22.58	13.51	4.89	0.81
	22	33.46	26.06	18.56	14.10	7.82
BasketballPass	27	40.51	26.73	17.34	10.57	4.85
(416 × 240)	32	46.54	26.96	16.49	7.18	2.83
	37	53.72	25.83	13.94	4.70	1.81
Average		58.33	18.26	13.35	7.45	2.61

**Table 3:** The distribution of the QTMT depth difference between adjacent frames (unit: %).

Sequences	QP	d = 0	d = 1	d = 2	d ≥ 3	d ≤ 1
FourPeople	22	46.8	25.8	14.1	13.3	72.6
	27	66.0	17.3	8.9	7.8	83.3
	32	73.6	15.0	6.1	5.3	88.6
	37	79.2	12.1	5.1	3.6	91.3
MarketPlace	22	22.0	34.5	25.1	18.4	56.5
	27	35.6	34.4	17.1	12.9	70.0
	32	44.3	32	13.8	9.9	76.3
	37	54.0	27.3	10.8	7.9	81.3
Average		52.7	24.8	12.6	9.9	77.5

**Table 4:** The distribution of the QT depth difference between adjacent frames (unit: %).

Sequences	QP	d = 0	d = 1	d = 2	d ≥ 3	d ≤ 1
FourPeople	22	65.1	27.7	6.6	0.6	92.8
	27	76.2	18.3	5.0	0.5	94.5
	32	81.7	15.0	3.0	0.3	96.7
	37	83.7	13.9	2.2	0.2	97.6
MarketPlace	22	42.2	50.2	7.2	0.4	92.4
	27	57.6	36.3	5.7	0.4	93.9
	32	67.1	28.5	4.2	0.2	95.6
	37	71.9	25.5	2.5	0.1	97.4
Average		68.2	26.9	4.6	0.3	95.1

**Table 5:** The distribution of the MT depth difference between adjacent frames (unit: %).

Sequences	QP	d = 0	d = 1	d = 2	d ≥ 3	d ≤ 1
FourPeople	22	53.5	29.4	11.0	6.1	82.9
	27	75.9	15.0	5.5	3.6	90.9
	32	81.6	11.9	4.0	2.5	93.5
	37	87.9	8.0	2.7	1.4	95.9
MarketPlace	22	30.3	39.8	21.0	8.9	70.1
	27	45.3	34.9	13.4	6.4	80.2
	32	51.8	32.7	10.4	5.1	84.5
	37	61.8	26.4	7.7	4.1	88.2
Average		61	24.8	9.5	4.7	85.8

**Table 6:** The probability that the MVD of a CU is 0 (unit: %).

	QP	22	27	32	37	Average
Sequences						
BasketballPass		96.5	97.3	97.8	98.6	97.5
FourPeople		87.1	91.9	92.7	93.6	91.3
MarketPlace		96.9	98.4	98.8	99.1	98.3
BasketballDrill		91.9	92.2	92.6	92.5	92.3

**Table 7:** Coding performance of proposed methods.

Class	Sequences	Early Skipping TT Partition		Early Terminating QTMT Partition		Proposed Overall Method	
		BDBR(%)	TS(%)	BDBR(%)	TS(%)	BDBR(%)	TS(%)
A1	Campfire	1.91	40.85	0.14	1.00	1.98	42.08
	FoodMarket4	0.11	13.21	0.05	9.36	0.19	21.10
	Tango2	0.59	16.14	0.56	7.94	0.98	21.06
A2	CatRobot1	0.30	10.46	0.46	6.30	0.86	18.85
	DaylightRoad2	0.61	16.37	0.53	2.23	1.15	18.88
	ParkRunning3	0.70	29.06	0.10	0.92	0.76	30.92
B	BasketballDrive	0.86	20.93	0.55	4.13	1.23	27.61
	BQTerrace	0.38	20.68	0.28	6.48	0.55	27.91
	Cactus	0.40	17.10	0.40	6.67	0.77	25.42
	MarketPlace	0.46	11.86	0.42	2.76	0.81	18.09
	RitualDance	1.29	26.22	0.31	2.57	1.65	31.79
C	BasketballDrill	0.88	21.64	0.48	8.05	1.30	30.12
	BQMall	0.76	14.91	0.71	9.03	1.35	25.25
	PartyScene	1.04	24.24	0.24	2.48	1.02	28.77
	RaceHorsesC	1.14	22.18	0.29	4.39	1.54	30.11
D	BasketballPass	0.31	3.92	0.89	12.04	0.99	18.63
	BlowingBubbles	0.37	8.27	0.69	12.10	1.07	21.88
	BQSquare	0.06	3.28	0.32	10.25	0.34	16.02
	RaceHorses	0.94	13.05	0.83	10.86	1.63	25.48
E	FourPeople	0.16	5.03	0.30	5.43	0.38	11.73
	Johnny	0.08	0.77	0.12	4.63	0.29	8.88
	KristenAndSara	0.15	0.48	0.16	5.35	0.44	9.57
	Average	0.61	15.48	0.40	6.14	0.97	23.19

**Table 8:** Coding performance comparison of fast inter CU partition methods.

Class	Sequences	[30]		[32]		Proposed Method	
		BDBR(%)	TS(%)	BDBR(%)	TS(%)	BDBR(%)	TS(%)
A1	Campfire	2.87	28.21	2.80	30.08	1.98	42.08
	FoodMarket4	1.41	32.21	1.59	42.90	0.19	21.10
	Tango2	3.75	29.29	3.68	34.05	0.98	21.06
A2	CatRobot1	4.18	30.12	5.59	30.62	0.86	18.85
	DaylightRoad2	4.84	29.63	4.43	29.20	1.15	18.88
	ParkRunning3	3.05	31.54	1.61	21.30	0.76	30.92
B	BasketballDrive	5.22	35.11	2.96	32.39	1.23	27.61
	BQTerrace	1.95	34.64	0.98	13.80	0.55	27.91
	Cactus	2.93	31.18	5.20	25.42	0.77	25.42
	MarketPlace	3.30	34.19	3.22	36.47	0.81	18.09
	RitualDance	5.49	38.53	2.97	31.23	1.65	31.79
C	BasketballDrill	1.74	15.30	1.59	24.38	1.30	30.12
	BQMall	0.31	8.29	2.35	22.41	1.35	25.25
	PartyScene	2.08	20.56	1.84	14.94	1.02	28.77
	RaceHorsesC	3.15	20.66	2.23	22.55	1.54	30.11
D	BasketballPass	10.10	24.26	1.56	21.18	0.99	18.63
	BlowingBubbles	2.26	29.25	2.29	16.97	1.07	21.88
	BQSquare	3.37	33.09	0.84	9.69	0.34	16.02
	RaceHorses	6.21	33.85	2.24	20.33	1.63	25.48
E	FourPeople	0.27	11.59	1.76	25.26	0.38	11.73
	Johnny	1.96	17.68	1.69	24.92	0.29	8.88
	KristenAndSara	1.87	15.18	2.11	26.21	0.44	9.57
	Average	3.29	26.56	2.52	25.29	0.97	23.19

## Data Availability

Not applicable.
